# Antimetastatic Action of Pentoxifylline, a Methyl Xanthine Derivative, Through its Effect on PKC Mediated Integrin Transport in B16F10 Melanoma Cells

**DOI:** 10.4021/wjon252e

**Published:** 2010-11-02

**Authors:** Aparna Ratheesh, Meenakashi Jain, Rajiv P Gude

**Affiliations:** a Advanced Centre for Treatment, Research & Education in Cancer (ACTREC), Tata Memorial Centre, Kharghar, Navi Mumbai, India-410210; c These authors contributed equally to this work

**Keywords:** Pentoxifylline, Integrin, Internalization, Recycling, Protein kinase C

## Abstract

**Background:**

Integrins are adhesion molecules known to regulate cellular processes like adhesion, migration and proliferation. At the same time role of integrin in progress of cancer metastasis is well established, increased integrin expression is reported to be linked to high metastasis potential of cells. Pentoxifylline a methyl xanthine derivative is a potent antimetastatic agent. Studies on the mechanism of inhibition of lung homing of B16F10 melanoma cells by PTX shows that it can inhibit cell- Extracellular Matrix adhesion, cell surface integrin expression as well as Protein kinase C activity. Previous study from our laboratory have shown PTX treatment can selectively inhibit the cell surface expression of α5 integrin in B16F10 cells without affecting its total cellular protein levels. Numerous studies have documented that differences in surface expression and distribution of integrins affects metastasis. The purpose of present study is to observe the effect of PTX on cellular distribution/ redistribution of integrins and to study the underlying molecular mechanism of PTX action.

**Methods:**

Integrin internalization and transport was observed using immunofluorescence confocal microscopy. PKC activity was determined using MBP4-14 as a substrate. Immunoprecipitation and western blotting was used to show association between PKC and α5 integrin, cell adhesion assay was performed using fibronectin/fibrinogen as substrate.

**Results:**

Immunofluorescence studies showed that PTX treatment caused a redistribution of α5 integrins from the plasma membrane to a perinuclear compartment where it colocalized with Transferrin receptor and Rab-11 GTPase. Rate of integrin internalization and recycling showed that PTX inhibited the recycling of α5 integrins from perinuclear recycling endosomes. PTX is reported to affect kinases; here we showed that PTX inhibited total PKC activity. Association between α5β1 integrin and PKC is studied using Immunoprecipitation which show that PTX affects α5β1 integrin associated PKC activity without affecting the levels of PKC. Studying the effect of delay in integrin recycling on cell functionality showed that it affects spreading of cells on fibronectin/fibrinogen.

**Conclusions:**

Data in the present study shows that PTX interferes with PKC activity bringing about a change in integrin distribution, and there by affecting the functionality of the cell. And this may possibly serve as one of the mechanisms for antimetastatic action of PTX.

## Introduction

Rising incidences of melanoma worldwide and high rate of mortality associated with it is attributed to its relentless growth of metastases. Metastasis is a complex cascade of events including adhesion, migration, angiogenesis, and invasion and the overall process has hitherto evaded almost all therapeutic modalities. Pentoxifylline (3, 7, dimethyl-1-5-oxohexyl xanthine), was used initially as a hemorreological agent in the treatment of peripheral vascular disease [1, 2]. This non-specific phosphodiesterase inhibitor, has also been shown to act as a potent anti-metastatic agent, elevate cAMP dependent Protein Kinase A activity [3], inhibit Tumor Necrosis Alpha (TNF-α) production [4] and inhibit Protein Kinase C activity [5].

PTX has been shown to inhibit IL-2 toxicity, preserve anti-tumor efficacy in patients with metastatic renal cell carcinoma [6]**,** prevent radiation-induced side-effects in women with breast cancer [7] and act as a potent antimetastatic agent, suppressing the metastasis rates in hepatocellular carcinomas [4]. Although the mechanism behind the antimetastic action of PTX has not been fully elucidated, studies from our laboratory have shown that the effects of PTX are mediated atleast in part through its effect on integrin expression and integrin mediated adhesion.

Integrins are heterodimeric transmembrane glycoproteins, which mediate communication between cells and the ECM. Integrin adhesion to proteins in the ECM acts as a central regulatory mechanism for a wide range of cellular processes including proliferation, differentiation, migration, and apoptosis. Aberrations in these processes lead to pathological conditions including cancer. Increased integrin expression has been shown to correlate with increased metastatic potential of many carcinomas [8-11] and for these reasons, studies on mechanisms of integrin regulation and agents targeting integrins are currently of interest.

Our studies on the mechanism of antimetastatic action of PTX show that this drug can inhibit secretion of matrix metalloproteases, invasion through extracellular matrix and cell - ECM adhesion [12]. Further studies on the effect of PTX on integrin expression showed that PTX treatment can selectively inhibit the cell surface expression of α5 integrin in cultured B16F10 cells without affecting the total cellular protein levels of these integrins [5]. Therefore, in this study, we looked at the effect of PTX on the sub cellular localization and rates of transport or distribution /redistribution of α5β1 integrins. It has been known that α5 integrins form heterodimers only with the β1 subunit [13]. Most integrin heterodimers constantly traffic between the plasma membrane and endosomal compartments in repeating endo/exocytic cycles which helps in rapid receptor turnover [14, 15]. Integrin transport has been shown to be regulated by PKC activity associated with the integrins [16], so we checked whether PTX has any effect on PKC expression and its activity associated with α5β1 integrins. Our data shows, for the first time, that Pentoxifylline can affect integrin distribution /redistribution (transport) and can inhibit the recycling of α5 integrins from perinuclear recycling endosomes. The data suggests that this inhibition is mediated through its effects on PKC activity. We also observed inhibitory effects of PTX on cell spreading, one of the contributing events in the tumor metastasis cascade.

## Materials and Methods

### Reagents and antibodies

Fibronectin, Leupeptin, Pepstatin, Aprotinin, Phenylmethanesulphonyl fluoride (PMSF), Myelin Basic Protein fragment 4-14, Protein Kinase A inhibitor fragment 14-2: myristoylated trifluoroacetate salt and Protein Kinase C Inhibitor, myristoylated were purchased from Sigma Aldrich, USA. g-32P Adenosine 5’ Triphosphate Triethylammonium Salt was purchased from Board of Radiation and Isotope Technology (BRIT), Hyderabad, India. NHS-SS-Biotin was purchased from Pierce, USA. Protein G Sepharose TM and PVDF were purchased from Amersham Biosciences, UK. Tarsons, India and NUNC, Denmark supplied the plastic ware for cell culture work.

Monoclonal antibodies against Integrins β1, α5 ,Transferrin receptor, Rab-4, Rab-11, β-tubulin, FITC-conjugated monoclonal anti-mouse antibodies against Integrin β1 , normal Armenian hamster IgG, PE-conjugated normal rat IgG1 and PE-conjugated normal rat IgG2a was purchased from Santa Cruz Biotechnology Inc, USA. Monoclonal anti-mouse antibody MAB 2575 against integrin α5β1, Cy 3 conjugated anti-rabbit IgG were purchased from Chemicon International, USA. R-PE-conjugated anti-mouse antibody against integrin α5 was purchased from Pharmingen, BD Biosciences, USA. HRPO conjugated anti-rat and anti-mouse and anti-rabbit IgG, TRITC conjugated anti-goat IgG were purchased from Sigma Aldrich, USA. FITC conjugated anti-rat IgG and anti-rabbit IgG was purchased from ICN Biomedical Inc, USA. Alexa 568 conjugated anti-rabbit and anti-mouse IgG were purchased from Molecular Probes, Invitrogen, USA.

Pentoxifylline was purchased from Sigma Aldrich, USA and dissolved in phosphate buffered saline (PBS) prior to use. IC 50 of PTX for 2 hours is 16 mM [12] and we have used PTX at a concentration of 1mM for the current study.

### Cell culture

B16-F10 cell lines were obtained from NCCS, Pune, India and maintained as described earlier [5]. For all the experiments, cells were grown to 80-90% confluency in IMDM containing 10% FBS.

### Immunofluorescence

Sterile coverslips containing control and PTX treated cells were fixed using 4% Paraformaldehyde and permeabilized using 0.1% Triton –X 100. After blocking in PBS containing 10% FBS, cells were incubated with the appropriate antibodies.

For visualizing α5 integrin, cells were incubated with primary antibody overnight at 4°C followed by FITC/Alexa 568/ Cy 3.5 conjugated secondary antibody. For dual staining with Transferrin receptor, Rab-4 and Rab-11, cells were incubated with antibodies for one hour followed by incubation with flurochrome conjugated secondary antibodies. For dual staining of integrins with Rab-4/Rab-11 antibodies, cells were permeabilized with 0.1% Saponin in 0.5% BSA for 15 minutes before staining.

Cells alone without any antibody staining and cells incubated with the flurochrome conjugated secondary antibodies alone (no primary antibody) were used as negative controls. Images were acquired at 63X using the LSM 510 META laser-scanning microscope from Carl Zeiss, Germany and analyzed using the software LSM 510. Five to six fields (each containing a minimum of four to five cells) were acquired for each coverslip and the experiments were repeated thrice. Representative images of projections of optical slices are shown here. Colocalization was estimated using the Overlap coefficient according to Manders which indicates an overlap of the signals and thus represents the true degree of colocalization [17]. The value of overlap coefficient ranges from 0 to 1.0 where a value of 1 indicates 100% colocalization and of zero indicates that there are no overlapping pixels.

### Internalization and recycling assay

The rates of internalization and recycling of integrins was measured as described [18].

#### Internalization

B16F10 cells were grown 90% confluent. Cells were then serum starved for 30 minutes in the presence or absence of Pentoxifylline. Cell surface was labeled with 0.2 mg/ml NHS-SS-Biotin at 4°C for 30 minutes, excess biotin was washed off with ice cold PBS and cells were then incubated with prewarmed IMDM at 37°C for the indicated time points (0, 2.5, 5, 7.5 and 10 minutes) to allow internalization. Internalization was carried out in the presence of 0.6 mM Primaquine phosphate, which prevents recycling. At each time point the cells were removed from 37°C and the biotin remaining on the cell surface was removed by incubating the cells with 20 mM Sodium 2-mercaptoethanesulphonate (MesNa) for 15 minutes on ice. MesNa was quenched by addition of 20 mM iodoacetamide (IAA) for 10 minutes. The cells were lysed (200 mM NaCl, 75 mM Tris, 15 mM NaF, 1.5 mM Na3VO4, 7.5 mM EDTA, 7.5 mM EGTA, 1.5% Triton X-100, 50 µg/ml leupeptin, 50 µg/ml aprotinin), protein concentrations were normalized and the levels of biotinylated integrins were measured by Capture ELISA.

#### Recycling assays

For recycling assays, post surface labeling, cells were transferred to prewarmed plain IMDM and Biotin was allowed to internalize to early endosomes and perinuclear recycling endosomes by incubation at 22°C for 20 minutes or 37°C for 30 minutes respectively. After internalization cells were returned to ice, biotin stripped off from the cell surface and the internalized fraction was allowed to recycle by incubation at 37°C for 0, 5, 7.5 and 10 minutes for recycling from early endosomes and 0, 5, 10 and 20 minutes for recycling from perinuclear recycling endosomes. At each time point the cells were returned to ice, biotin from the recycled proteins was stripped off by MeSNa. Biotinylated integrins were assessed by Capture ELISA.

### Capture-ELISA

Maxisorb 96-well plates (Nunc) coated overnight with 5 µg/ml appropriate anti-integrin antibodies were blocked in PBS containing 0.05% Tween-20 (PBS-T) with 5% BSA for 1 hr at room temperature. Integrins were captured by overnight incubation of 50µl cell lysates at 4°C followed by incubation with streptavidin-conjugated horseradish peroxidase. Following further washing, biotinylated integrins were detected by a chromogenic reaction with ortho-phenylenediamine.

### Immunoprecipitation

Cell lysate was prepared as described [5]. 2mg of the cell lysate (from control and PTX treated cells) was incubated with Protein-G Sepharose TM beads for 1 hour for preclearing. The supernatants were then incubated with antibody against α5β1 integrins overnight at 4°C with constant rotation. These were further incubated with Protein-G Sepharose TM beads for 2 hours at 4°C with rotation. The beads were washed with lysis buffer and PBS. The bead fraction was then divided into two.

One fraction was boiled for 5 minutes in Laemmli buffer and the supernatant containing the integrins and associated proteins was loaded onto an 8% SDS gel. The proteins on the gel were transferred onto a polyvinylidene difluoride (PVDF) membrane and immunoblotting was performed for α5β1 integrins and PKC. The second fraction was analyzed for integrin associated Protein Kinase C activity using radioactive PKC assay.

### Western Blotting

The assay was carried out as described previously [5].

### Measurement of PKC activity

PKC assays were carried out as described previously [5].

### Cell spreading assay

Sterilized tissue culture coverslips were incubated overnight with 5 µg/ml ECM substrates at 4°C and washed extensively with PBS before use. B16F10 cells were grown to 80-90% confluency, the cells harvested using Saline EDTA and plated onto substrate coated coverslips. Cells were then incubated for a further 30 minutes before fixation and stained for F-actin and integrin.

### Statistical Analysis

Results are representative of three or more experiments. Quantitative values are expressed as mean ± S.E. Statistical measurements were made using SPSS software version 14. Significance for differences between the samples was measured by student’s t-test or by One Way ANOVA (equal variances assumed) with Dunnett t-test.

## Results

### Pentoxifylline alters the localization of α5 integrins in melanoma cells

**Figure 1:**
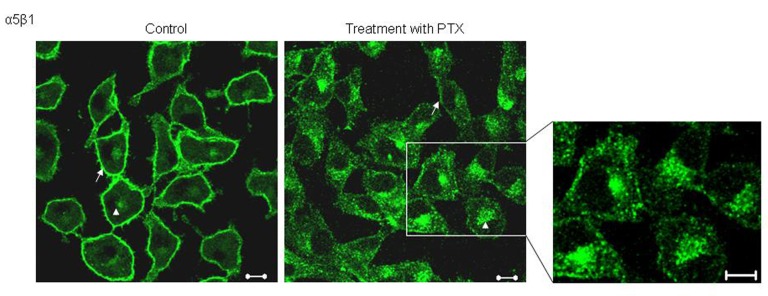
Effect of PTX on the localization of α5 integrins. Untreated and PTX treated cells, fixed and probed with an antibody against α5 integrins and visualized by FITC conjugated secondary antibody. Representative images are shown here. Scale bar, 10 µm.

Studying the localization of α5 integrins, we observed that in untreated cells, α5 integrins predominantly stained the plasma membrane (Fig. 1, arrows) along with some perinuclear staining. Post PTX treatment, B16F10 cells showed changes in the pattern of α5 staining; there was an increase in the amount of intra cellular, perinuclear staining (Fig. 1). We had previously shown that PTX treatment decreases the cell surface expression of *α*5 integrins by around 40% and present data shows that PTX alters the localization of *α*5 integrins.

### Post PTX treatment, α5 integrins colocalizes with Rab-11 and transferrin receptor - markers of recycling endosomes

**Figure 2:**
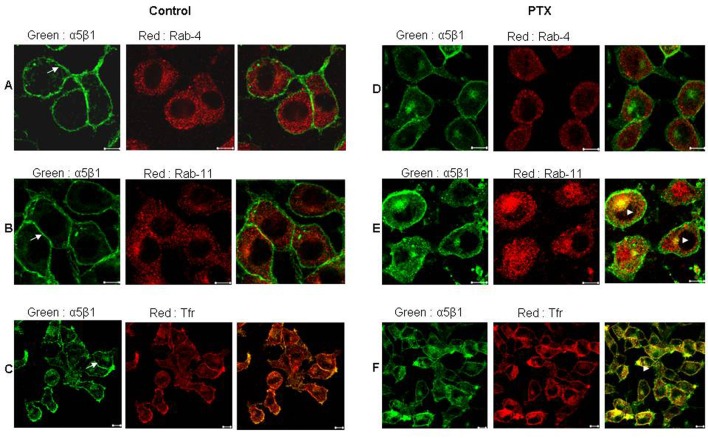
Post PTX treatment α5 integrins colocalized with Transferrin receptor and Rab 11 GTPase. Control (A, B and C) and PTX (D, E and F) treated F10 cells stained for α5 integrins (Green) and Rab 4 GTPase (A and D), Rab 11 GTPase (B and E) and Transferrin receptor (C and F) in Red. Yellow indicates colocalization of the two flurophores. Representative images are shown here. Scale bar, 10µm.

Since PTX treated cells showed altered localization of α5 integrins, the exact location of these integrins was investigated using markers to various endosomal compartments (Fig. 2). In untreated cells there was very minimal colocalization of α5 integrins with both Rab-4, a marker of early endosomes (Fig. 2A) and Rab-11, a marker of recycling endosomes (Fig. 2B). Post PTX treatment there was no increase in colocalization of α5 integrins with Rab-4 (Fig. 2D) which is also reflected in the lack of change in the values of mean overlap coefficients (Table 1). However, α5 integrins showed a marked increase in colocalization with Rab 11 and endogenous Transferrin receptor, both of which are markers for perinuclear recycling endosomes (Fig. 2E and 2F, arrowheads) and this increase in colocalization was reflected in the increase in the values of mean overlap coefficients suggesting that post PTX treatment α5 integrins selectively accumulate at the perinuclear recycling endosomes.

**Table 1:** Average Overlap Coefficients According to Manders Before and After PTX Treatment, Including Colocalization of α5 Integrin With Rab-4, Rab-11 and Transferring Receptor

	Control	PTX
α5 integrin/Rab-4	0.4 ± 0.228	0.52 ± 0.671
α5 integrin/Rab-11	0.5 ± 0.864	0.87 ± 0.72
α5 integrin/Transferring receptor	0.67 ± 0.023	0.91 ± 0.024

### PTX treatment decreases recycling of integrins from Rab 11 positive endosomes

**Figure 3:**
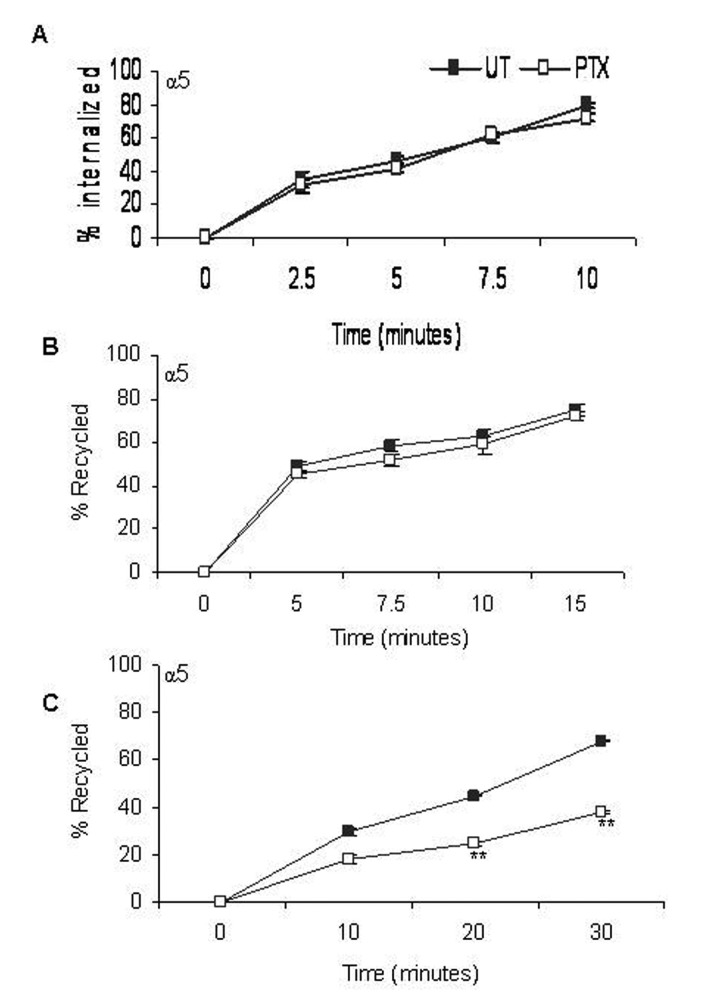
Effect of PTX on rates of α5 integrin distribution /redistribution. Rates of internalization (A), recycling from early endosomes (B) and recycling from perinuclear endosomes (C) of α5 integrins were measured. Closed squares represent rates of integrin transport in untreated cells and open squares represent that of PTX treated cells. Results represent the mean ± s.e.m of three independent experiments. **P < 0.01 vs untreated control (Students t-test).

Integrin internalization was studied by surface labeling F10 cells with membrane impermeable Biotin for various time points in the presence of the recycling inhibitor, Primaquine. In untreated cells, approximately 40% of the cell surface α5 integrins were internalized in 5 minutes and by the end of 10 minutes, a steady state was reached (Fig. 3A), whereas PTX treated cells showed no change in the rates of internalization of α5 integrins.

Integrin recycling was studied using NHS-SS-Biotin surface labeling. Approximately 50% of the α5 integrin, which had accumulated in the early endosomes, had recycled back within 5 minutes (Fig. 3B). Post PTX treatment, there was no decrease in the rate of recycling of α5 integrins from the early endosomes. Recycling from the perinuclear endosomes was slower and only about 35% of the α5 integrins had recycled back in 5 minutes. We reported a significant decrease in the rate of recycling of α5 (45% + 1.25), P < 0.001, students t-test) integrins from perinuclear recycling endosomes (Fig. 3C).

**Figure 4:**
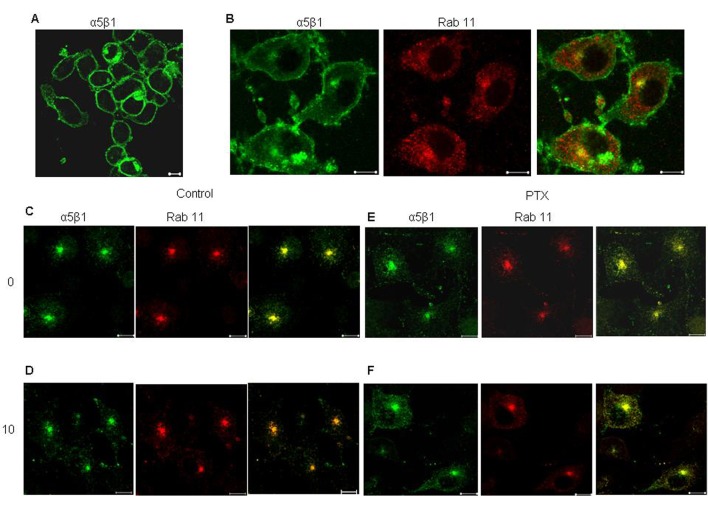
Effect of PTX on recycling of α5 integrin from perinuclear recycling endosomes. Surface α5β1 integrins, labeled with anti mouse α5 integrin antibody for 30 minutes at 4°C (A), allowed to internalize at 37°C for 30 minutes (B. The antibody remaining on the surface was stripped off (C and E, 0 minutes) and the internalized fraction was allowed to recycle by incubation at 37°C 10 minutes (D and F). Yellow indicates colocalization of the two flurophores. Representative images are shown here. Scale bar, 10 µm.

The effect of PTX on the recycling of α5 integrins was confirmed by immunofluorescence. In untreated cells, the amount of integrin remaining inside the Rab11 positive endosomes after 10 minutes of recycling was considerably lower as compared to the amount of integrin before recycling (Fig. 4). This was reflected in the values of average overlap coefficient, which decreased from 0.92 + 0.447 to 0.62 + 0.697. However, PTX treated cells showed no signs of decrease (average overlap coefficient at 0 minutes was 0.89 + 0.52 and at 10 minutes was 0.9) in the intracellular content of labeled integrins suggesting that PTX cause accumulation of α5 integrins at the recycling endosomes.

### PTX inhibits integrin associated PKC activity

**Figure 5:**
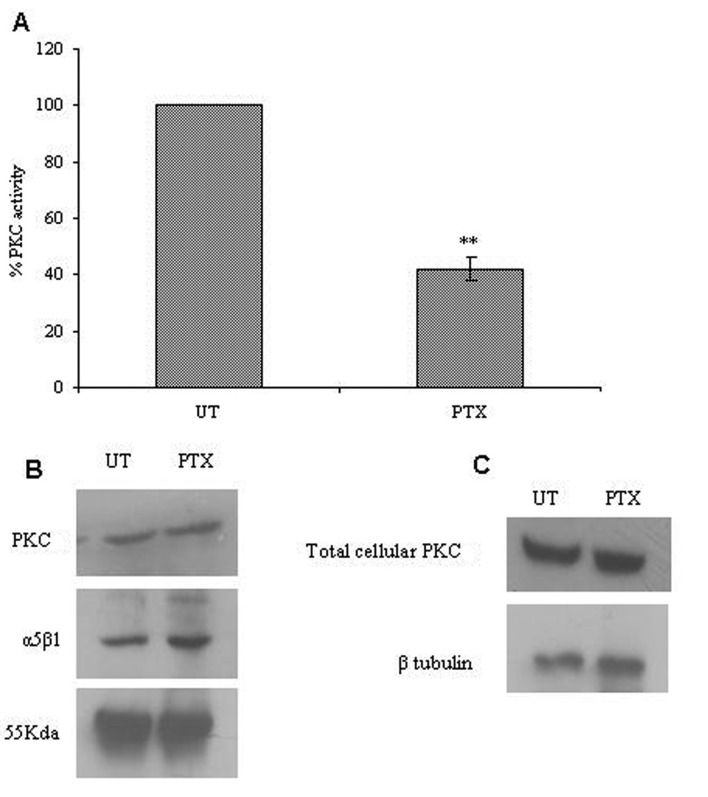
Effect of PTX on integrin associated PKC expression and activity. The α5β1 integrin was immunoprecipitated from control and PTX treated cells, and used to assay for integrin associated PKC activity (A) or analyzed for the presence of α5β1 integrins and PKC by western blotting (B). Western blotting was done for PKC in total cell lysates obtained from control and PTX treated cells (C). The experiment was repeated thrice and representative images are shown here. Graph A represent the mean ± s.e.m of three independent experiments. **P < 0.01 vs untreated control (Students t-test).

We studied the effects of PTX on PKC activity associated with α5β1 integrins. Post PTX treatment there was a 50% reduction in the PKC activity associated with α5β1 integrins (P < 0.001, students t-test) but there was no change in associated PKC levels (Fig. 5A, B). Total cellular lysates showed no changes in the protein levels of PKC and α5β1 integrins on PTX treatment.

### PTX inhibits cell spreading

**Figure 6:**
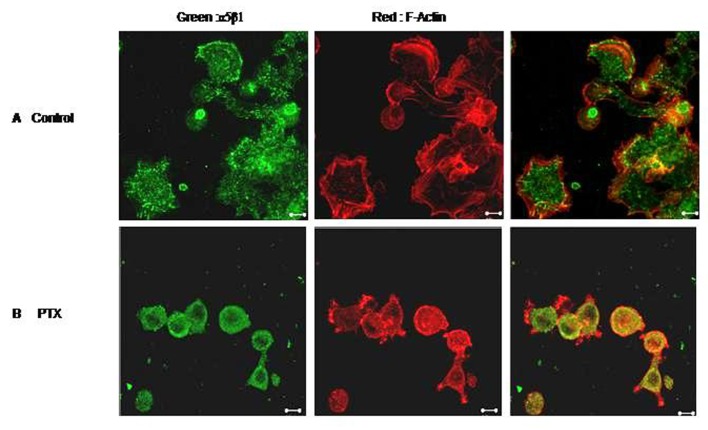
Effect of PTX on cell spreading. F10 cells were allowed to spread on Fibronectin coated plates in the presence or absence of Pentoxifylline. Control (A) and PTX treated (B) cells were then fixed, permeabilised and stained for F-Actin (red) and α5 integrin (green). Representative images are shown here. Scale bar, 10 µm.

We next checked the effect of PTX on cell spreading. Untreated B16F10 cells spread well on Fibronectin /Fibrinogen coated plates within 30 minutes. Cells showed development of numerous lamellopodial extensions (Fig 6). F-Actin staining was strong on the membranes where actin formed thick bundles along the lamellopodial margins. PTX treated cells, on the other hand, could not spread as effectively. The α5β1 integrins also accumulated in perinuclear endosomes-like vesicles with diminished membrane staining.

## Discussion

Present study is aimed to observe the molecular mechanism of Pentoxifylline action. The purpose is to specifically determine if the change in surface expression of the integrins after Pentoxifylline treatment is due to its effect on integrin distribution /redistribution.

Pentoxifylline, a phosphodiesterase inhibitor has been shown to affect metastasis rates in several cancer types [4, 19]. Studies from our laboratory have shown that Pentoxifylline inhibits the lung homing of B16F10 melanoma cells [4] and that this inhibition is mediated atleast in part by its effect on integrin receptors and integrin mediate adhesion. We have also observed that Pentoxifylline can inhibit the cell surface expression of α5β1 integrins, with no change in the total cellular protein level of the integrin in the cell.

It has been know for more than a decade now that certain integrin heterodimers constantly traffic between the plasma membrane and intra cellular endosomal compartments in a repeating endo/exocytic cycle which helps in rapid receptor turnover [4, 20] Initial studies on integrin internalization/recycling had given rise to models which suggested an increased requirement for integrin internalization at the rear end, after which these integrin containing vesicles are transported to the leading lamellopodial edges of a migrating cell.. Integrins can recycle, either from the early Endosomes (EEs) in a short loop of recycling or from the perinuclear recycling endosomes (PNERs) in a long loop of recycling [4]. In the present study we looked at the subcellular localization as well as the rate of recycling of α5β1 integrins after PTX treatment. Here we show that in basal conditions, the phosphodiesterase inhibitor, Pentoxifylline can cause selective inhibition in recycling of α5β1 integrins from perinuclear recycling endosomes.

We observed that on PTX treatment, α5β1 integrins accumulated in intracellular perinuclear compartments. We also observed cytoplasmic staining different from untreated cells, where α5 integrins predominantly stained the plasma membrane. Further analysis, after staining with Rab4, (marker for early endosome,EEs), Rab11 and Transferrin (marker for perinuclear recycling endosomes, PNREs) showed that, post PTX treatment there was accumulation of these integrins in the perinuclear recycling compartment, where integrins colocalized with Rab-11 and Transferrin receptor. Accumulation of α5 integrins in PNREs could be a result of high rates of internalization from the plasma membrane or decreased recycling from the endosomes. Hence, we looked at the effects of PTX on rates of integrin internalization and recycling. Quantitative assessment of the integrin transport revealed that PTX treatment inhibited the recycling of α5 integrins from PNREs.

Regulated associations of kinases with integrins have been shown to influence expression and transport. Protein kinase B (PKB/Akt) has been shown to be involved in regulating transport of β1 integrins from PNREs [20-22]. Recycling of β1 integrins from PNREs have been shown to be regulated by Vimentin phosphorylation by PKCε [23, 24] and PKD1.

The β1 integrin has been shown to associate with PKCα resulting in regulation of its internalization [25]. Previously, data from our laboratory showed that PTX can inhibit cellular PKC activity [5, 26]. Hence, we looked at the levels of PKC activity associated with α5β1 integrins to check the possible role of PKC in the inhibition of integrin transport by PTX. We observed that, post PTX treatment, there was a decrease in PKC activity associated with α5β1 integrins but not in its total protein levels associated with the said integrins. Our data suggests that PTX inhibits the recycling of α5β1 by decreasing the PKC activity associated with this integrin. In view of the present data we can assume the hypothesis that PTX brings about an inhibition in integrin recycling by an inhibition in integrin associated PKC activity.

It has been reported that integrins in the cell have direct effects on cell spreading and motility [27-29]. Inhibition of endo/exocytic recycling of αvβ3 integrins from early endosomes to cell surface impairs cell adhesion and spreading [18]. In our study we found that, B16F10 melanoma cell spread well on fibronectin (specific substrate for α5β1 integrin). Treated cells didn’t spread well with integrin α5β1 being localized in perinuclear recycling endosomes. PTX treated cells also showed actin depolimerization (PTX is a known actin depolimerizer) [12]. PTX also had a significant effect on the cell motility of the melanoma cells. No changes in the cytoskeletal tubulin network in cells were observed on PTX treatment (data not shown).

Taken together our data suggests that PTX can inhibit kinase associated integrin recycling, which affects important events (cell spreading, adhesion and motility) in the metastasis cascade. Studies with antimetastatic agents like Pentoxifylline can offer interesting insights into cancer therapy.

## Abbreviations

PTX: Pentoxifylline
ECM: Extracellular Matrix
PKC: Protein kinase C
TFr: Transferrin receptor
PNERs: Perinuclear Recycling Endosomes
Ees: Early Endosomes
